# The effect of tightened compulsory admission laws on length of stay in emergency department for suicide attempters

**DOI:** 10.1186/s12913-026-14474-6

**Published:** 2026-04-10

**Authors:** Arum Choi, Joung-Ho Han, Sukil Kim

**Affiliations:** 1 Department of Preventive Medicine and Public Health, College of Medicine, The Catholic University of Korea, Seoul, Republic of Korea; 2 Department of Internal Medicine, Chungbuk National University College of Medicine & Chungbuk National University Hospital, Cheongju, Republic of Korea; 3 Present Address: Department of Radiology and Research Institute of Radiology, Asan Medical Center, University of Ulsan College of Medicine, Seoul, Republic of Korea

**Keywords:** Suicide attempt, Emergency departments, Length of stay, Mental illness, Mental health welfare act

## Abstract

**Background:**

Suicide remains a major public health concern in South Korea. The emergency department (ED) is key in initial care for suicide attempters. In May 2017, South Korea implemented the Mental Health Welfare Act (MHWA), which strengthened procedural requirements for compulsory psychiatric admission. These changes may have affected admission processes and ED length of stay (LOS). This study evaluated the association between implementation of the MHWA and ED LOS for suicide attempters.

**Methods:**

We conducted a retrospective observational study using the National Emergency Department Information System (NEDIS) database from 2016 to 2019. Patients aged ≥ 20 years who presented to level I or II emergency medical centers with intentional self-harm (ICD-10 codes X60–X84) and were admitted or transferred were included. To account for both the Mental Health Welfare Act (MHWA; May 30, 2017) and a subsequent Emergency Medical Services Act (EMSA) amendment (December 3, 2017), the study period was divided into three phases. Median ED LOS was compared using the Kruskal–Wallis test with Dunn’s post hoc analysis, and Cox proportional hazards regression was performed adjusting for age and sex.

**Results:**

A total of 45,124 suicide attempters were analyzed. The median ED LOS for patients admitted to psychiatric departments was 7.4 h before the MHWA and 7.8 h after its implementation (*P* = 0.79). A statistically significant increase in ED LOS was observed in the period after both the MHWA and EMSA amendment (median 9.15 h; *P* < 0.05). In Cox regression analysis, no significant change was observed immediately after the MHWA (HR = 1.03, *P* = 0.60), whereas ED LOS was longer in the later period (HR = 0.91, *P* < 0.05).

**Conclusion:**

Implementation of the MHWA was not associated with a statistically significant immediate change in ED LOS for suicide attempters. The increase in ED LOS observed during the later study period likely reflects the combined influence of concurrent policy changes and broader system-level factors rather than the MHWA alone.

**Supplementary Information:**

The online version contains supplementary material available at 10.1186/s12913-026-14474-6.

**Table:** 

Text box 1. Contributions to the literature
• This study evaluates the association between South Korea’s 2017 Mental Health Welfare Act (MHWA) and emergency department (ED) length of stay for suicide attempters using a nationwide database.
• It demonstrates that the MHWA was not associated with a statistically significant immediate change in ED length of stay.
• The observed increase in ED length of stay during the later study period suggests that the operational effects of mental health legislation may reflect the combined influence of concurrent policy changes and broader system-level factors.
• These findings highlight that the operational effects of mental health legislation in emergency settings may depend on broader policy environments and healthcare system capacity.

## Background

Suicide is a major public health concern worldwide and remains a serious social issue in South Korea. The age-standardized suicide rate per 100,000 persons was 24.1 in 2020, the highest among the Organization for Economic Co-operation and Development (OECD) countries [1]. According to the cause of death statistics published by the Korean Statistical Information Service (KOSIS), the suicide mortality rate was 26.0 per 100,000 persons in 2021, an increase of 0.3 (1.2%) from the previous year. Suicide is also a leading cause of death among individuals aged 10 to 30 [2]. 

The emergency department (ED) is often one of the first medical institutions encountered by suicide attempters [3, 4], serving as a critical point for the medical evaluation and the initiation of mental health services. People with a history of suicide attempts are at higher risk of reattempt within one year compared to those who have never attempted [5, 6]. Therefore, developing appropriate evaluation and treatment strategies for these patients at the time of ED presentation is crucial.

Compulsory admission is permitted in many countries for individuals at high risk of suicide to prevent self-harm or harm to others, ensure patient safety, and provide treatment [7, 8]. Common criteria for involuntary admission include the presence of mental illness, risk of self-harm or harm to others, and necessity of treatment [9, 10]. However, the use of compulsory admission remains controversial. While it can be a life-saving intervention for suicidal individuals, studies also demonstrated potential negative consequences, including damage to the therapeutic relationship, psychological trauma, perceived coercion, and subsequent treatment avoidance [11, 12]. Furthermore, the evidence supporting the suicide-preventive effectiveness of compulsory admission remains limited, and one of the studies reported that involuntary hospitalization may not reduce, and could potentially increase, subsequent suicide risk [13]. 

In this context, South Korea revised its Mental Health Act to the Mental Health Promotion and Mental Patient Healthcare Service Act (abbreviated as the Mental Health Welfare Act, MHWA), which has been in effect since May 30, 2017 [14]. Previously, a person with mental illness who posed a risk of harming themselves or others could be involuntarily hospitalized with the approval of only two legal guardians and one psychiatrist at the admitting institution. However, the MHWA substantially tightened these criteria: it now requires a secondary diagnosis by an external psychiatrist affiliated with a public or designated institution for involuntary admissions extending beyond two weeks [15, 16]. In practice, the external psychiatrist must either visit the admitting hospital or the patient must be transported to the designated institution for evaluation. Given the limited availability of qualified public hospital psychiatrists, particularly during nights and weekends, this requirement has created significant logistical challenges in practice [16]. 

These regulatory changes may have implications for the management of suicide attempters in the ED. While most psychiatric care is effectively managed in outpatient settings, patients presenting to the ED following a suicide attempt often require acute inpatient hospitalization for immediate safety and psychiatric stabilization [17]. When a suicide attempter presents to the ED and psychiatric admission is deemed necessary, the additional procedural requirements under the MHWA may delay the admission process, thereby prolonging ED length of stay (LOS). Furthermore, the revised Act allows patients to be discharged at any time upon request [18], which further complicates the ability of clinicians to secure necessary and timely inpatient care for these high-risk individuals.

Therefore, the purpose of this study was to investigate the potential impact of the MHWA on ED LOS for suicide attempters admitted to psychiatric departments, using a national database of ED visits.

## Methods

### Study design and data source

We conducted an retretrospective observational study using the National Emergency Department Information System (NEDIS) database, which contains both clinical and administrative data of patients who have visited EDs across the country.

### Study subjects

The study population included patients aged 20 years or older who visited EDs for intentional self-harm or suicide attempts between 2016 and 2019. Emergency medical institutions in Korea are classified into three levels according to function and capacity of the hospital. Level I is regional emergency medical centers (EMC), level II is local EMCs, and level III is local emergency medical institutes (EMI) [19]. Since data on intent to visit EDs are only available at the center level, only patients who visited level I or II facilities after self-harming or suicide attempts were included. Patients diagnosed with injury due to intentional self-harm (X60 ~ X84) based on the International Classification of Diseases, Tenth Edition (ICD-10) [20] were included. Patients who died or lacked information were excluded. The final analysis was performed on patients who were transferred or hospitalized with emergency treatment results.

### Variables

We investigated patient gender, age, hospital type, onset date and time, injury mechanism, consciousness state, visit time, arrival method, Korean Triage and Acuity System (KTAS) severity classification, emergency treatment outcomes, and final treatment results that were transmitted to the NEDIS. A psychiatric department admission was defined as cases in which the psychiatric department was the primary care department responsible for the patient’s care after visiting the ED. The ED LOS for admitted patients was defined as the time difference between arrival time and the physical departure of the patient from the ED treatment area.

### Statistical analysis

We performed the Student’s *t*-test or Mann-Whitney U test for continuous variables and the Chi-squared test or Fisher’s exact test for categorical variables to compare the characteristics of patients who visited EDs before and after the implementation of the law.

During the study period, an amendment to the Emergency Medical Services Act (EMSA), implemented on December 3, 2017, restricted patient stays in the ED to a maximum of 24 h to address ED overcrowding [21]. Because the MHWA (May 30, 2017) and the EMSA amendment were implemented only six months apart, we divided the study period into three phases to distinguish their respective effects: Period 1, before the MHWA (January 1, 2016–May 29, 2017); Period 2, after the MHWA but before the EMSA amendment (May 30–December 2, 2017); and Period 3, after the EMSA amendment (December 3, 2017–December 31, 2019) (Supplementary Fig. 1).

The Kruskal-Wallis test and Dunn’s multiple comparisons test were used to compare the median ED LOS among those periods. Cox regression analysis adjusted for age and sex was used to investigate the differences in ED LOS in the three periods. Data from the other departments were the same analyzed to compare the results. Because the COVID-19 pandemic significantly changed ED utilization and hospitalization procedures, only data before the pandemic were included in the analyses.

All statistical analyses were performed using R version 4.0.0 (R Foundation for Statistical Computing, Vienna, Austria) and SAS version 9.4 (SAS Institute Inc., Cary, NC, USA).

## Results

### Characteristics of hospitalized suicide attempt patients based on emergency treatment outcome

**Fig. 1:**
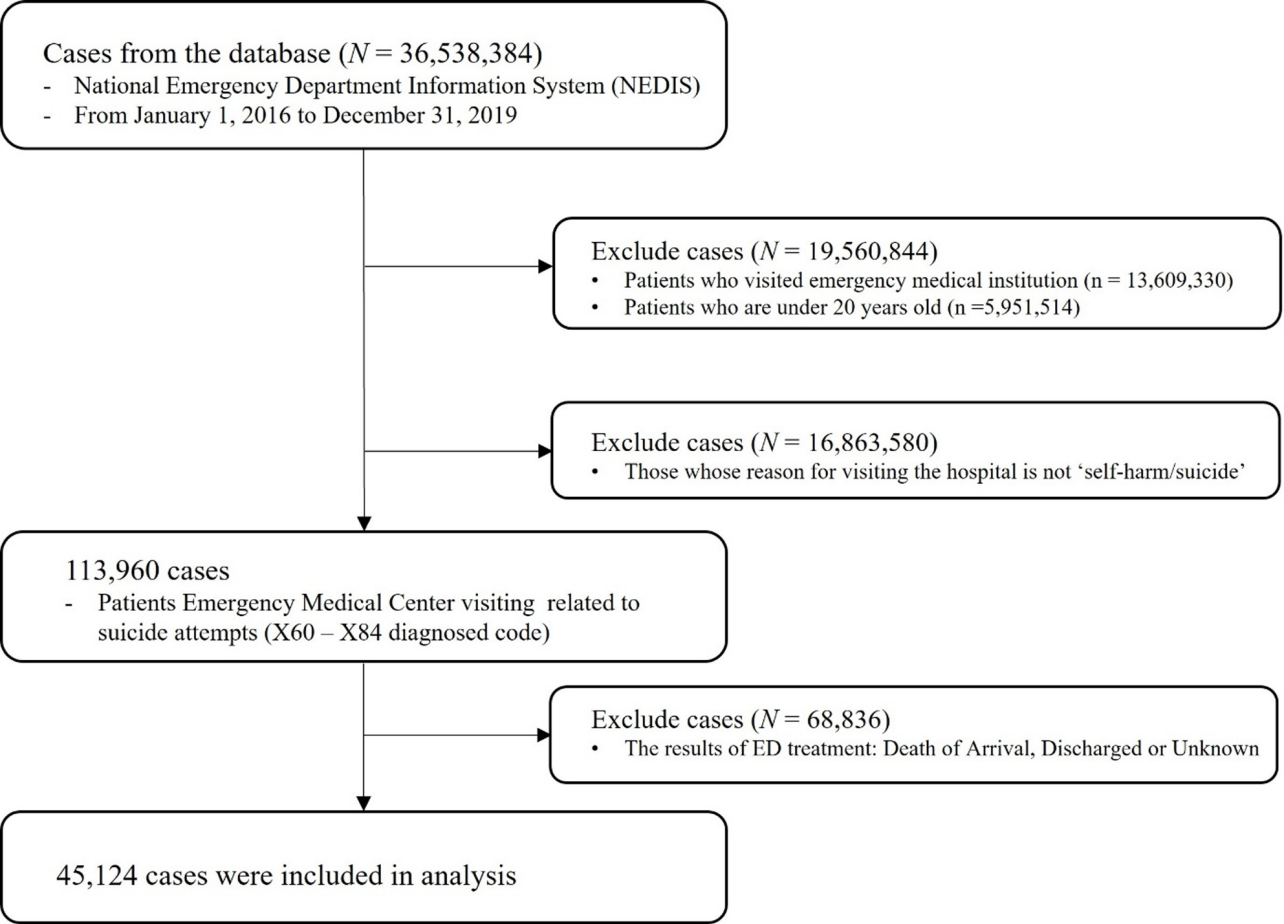
Flow chart of case selection

A total of 36,538,384 patients visited EDs during the study period. We selected patients who visited EMCs and were over 20 years old. After excluding patients whose emergency treatment outcomes were death, discharge, or unknown, 45,124 patients whose emergency treatment results were either referral or hospitalization were finally analyzed (Fig. 1).

Only KTAS classification was significantly different between suicide attempters admitted to psychiatric departments through EDs before and after the implementation of the Mental Health Welfare Act. The number of severe patients (KTAS Lv 1, 2) decreased after the MHWA was implemented, whereas the proportion of intermediate patients (KTAS Lv 3,4) increased (*P* < 0.01). There was no difference in emergency medical outcomes (admission to general ward, ICU, or transfer) or final disposition (discharge, self-discharge, or transfer) (Table 1).

**Table 1:** Comparison of psychiatric and non-psychiatry admissions of suicide attempters visiting emergency medical centers (EMCs) before and after implementation of the Mental Health Welfare Act

Variables	Admission Department					
	Psychiatry			Other		
	(*N* = 2,756)			(*N* = 42,368)		
	Before enforcement	After enforcement	*p*	Before enforcement	After enforcement	*p*
	(*N* = 956)	(*N* = 1800)		(*N* = 13,221)	(*N* = 29,147)	
Gender, n (%)			0.619			0.083
Female	539 (56.4%)	1034 (57.4%)		6695 (50.6%)	15,026 (51.6%)	
Age, n (%)			0.303			0.926
Young 20–64	647 (67.7%)	1254 (69.7%)		6759 (51.1%)	14,885 (51.1%)	
Old 65+	309 (32.3%)	546 (30.3%)		6462 (48.9%)	14,262 (48.9%)	
Hospital type, n (%)			0.279			< 0.01
District EMC	402 (42.1%)	797 (44.3%)		5123 (38.7%)	13,311 (45.7%)	
Regional EMC	554 (57.9%)	1003 (55.7%)		8098 (61.3%)	15,836 (54.3%)	
Time of suicide attempt, n (%)			0.795			-
06:00–18:00	485 (50.7%)	924 (51.3%)		6482 (49%)	14,289 (49%)	
18:00–06:00	471 (49.3%)	876 (48.7%)		6739 (51%)	14,858 (51%)	
Time from attempt to EMC visit			0.020			< 0.01
hours, median [IQR]	1.8 [ 0.8; 6.8]	2.0 [ 0.9; 8.2]		1.2 [ 0.6; 3.8]	1.3 [ 0.7; 3.9]	
Suicide attempt methods, n (%)			0.086			< 0.01
Cutting	206 (21.5%)	430 (23.9%)		1583 (12%)	3063 (10.5%)	
Drowning	16 (1.7%)	34 (1.9%)		111 (0.8%)	189 (0.6%)	
Falling	35 (3.7%)	35 (1.9%)		388 (2.9%)	884 (3%)	
Hanging	87 (9.1%)	161 (8.9%)		681 (5.2%)	1648 (5.7%)	
Poisoning	509 (53.2%)	932 (51.8%)		9701 (73.4%)	21,795 (74.8%)	
Others	103 (10.7%)	208 (11.5%)		757 (5.8%)	1568 (5.4%)	
Mental status at EMC entrance, n (%)			0.618			< 0.01
Alert	714 (74.7%)	1379 (76.6%)		6840 (51.7%)	14,343 (49.2%)	
Responds to vocal stimuli	171 (17.9%)	290 (16.1%)		2929 (22.2%)	6998 (24%)	
Responds to pain	67 (7%)	121 (6.7%)		2619 (19.8%)	5973 (20.5%)	
Unresponsive	4 (0.4%)	10 (0.6%)		820 (6.2%)	1833 (6.3%)	
Shift at arrival, n (%)			0.75			0.895
Day (8:00–16:00)	247 (25.8%)	484 (26.9%)		3176 (24%)	6946 (23.8%)	
Evening (16:00–00:00)	482 (50.4%)	908 (50.4%)		6777 (51.3%)	14,954 (51.3%)	
Night (00:00–8:00)	227 (23.7%)	408 (22.7%)		3268 (24.7%)	7247 (24.9%)	
Mode of arrival, n (%)			0.423			< 0.01
Self-referred	790 (82.6%)	1522 (84.6%)		10,585 (80.1%)	23,853 (81.8%)	
Outpatient department	5 (0.5%)	14 (0.8%)		15 (0.1%)	26 (0.1%)	
Referred from clinic	160 (16.7%)	262 (14.6%)		2616 (19.8%)	5263 (18.1%)	
Triage level (KTAS) n (%)			< 0.01			< 0.01
1	19 (2%)	22 (1.2%)		1257 (9.5%)	2494 (8.6%)	
2	408 (42.7%)	547 (30.4%)		6491 (49.1%)	12,599 (43.2%)	
3	374 (39.1%)	917 (50.9%)		4052 (30.6%)	11,431 (39.2%)	
4	144 (15.1%)	296 (16.4%)		1335 (10.1%)	2519 (8.6%)	
5	11 (1.2%)	16 (0.9%)		59 (0.4%)	98 (0.3%)	
ED LOS			0.085			< 0.01
hours, median [IQR]	7.4 [ 4.2;15.2]	8.8 [ 4.4;15.6]		3.5 [ 2.0; 7.6]	3.4 [ 2.0; 6.8]	
Emergency medical outcome, n (%)			0.103			< 0.01
Admission to GW	653 (68.3%)	1247 (69.3%)		3770 (28.5%)	8887 (30.5%)	
Admission to ICU	52 (5.4%)	86 (4.8%)		6733 (50.9%)	14,489 (49.7%)	
Admission to OR	5 (0.5%)	27 (1.5%)		693 (5.2%)	1281 (4.4%)	
Transfer to another hospital	246 (25.7%)	440 (24.4%)		2025 (15.3%)	4490 (15.4%)	
Final disposition, n (%)			0.166			< 0.01
Discharged	518 (54.2%)	988 (54.9%)		7294 (55.2%)	15,297 (52.5%)	
Self-discharged	138 (14.4%)	295 (16.4%)		1833 (13.9%)	5000 (17.2%)	
Left the hospital	0 (0%)	4 (0.2%)		9 (0.1%)	8 (0%)	
Expired	4 (0.4%)	2 (0.1%)		18 (0.1%)	53 (0.2%)	
Transfer to other hospital	23 (2.4%)	41 (2.3%)		621 (4.7%)	1310 (4.5%)	

EMC: emergency medical center; KTAS: Korean Triage and Acuity Scale, ED LOS: emergency department length of stay, GW: general ward, ICU: intensive care unit, OR: operation room

**Table 2:** Comparison of median ED LOS before and after law revision

(A) Psychiatry department				
Variables	Median [IQR]	*P* ^*^	Post-hoc test	
			Variables	*P* ^+^
Period 1 (*n* = 956)	7.40 [4.20;15.30]	< 0.05	Period 1 vs. Period 2	0.79
Period 2 (*n* = 354)	7.80 [3.90;14.70]		Period 1 vs. Period 3	< 0.05
Period 3 (*n* = 1,448)	9.15 [4.50;15.90]		Period 2 vs. Period 3	< 0.05

(B) Other departments				
Variables	Median [IQR]	*P* ^*^	Post-hoc test	
			Variables	*P* ^*+*^
Period 1 (*n* = 13,195)	3.50 [2.00;7.60]	< 0.05	Period 1 vs. Period 2	0.13
Period 2 (*n* = 5,597)	3.40 [2.00;7.10]		Period 1 vs. Period 3	< 0.05
Period 3 (*n* = 23,576)	3.40 [2.00;6.80]		Period 2 vs. Period 3	-

ED: emergency department; IQR: interquartile range

Period 1 was ED visits before the implementation of the Mental Health Welfare Act. Period 2 was ED visits after the implementation of the Mental Health Welfare Act. Period 3 was ED visits after the implementation of the modified Emergency Medical Services Act

The Mental Health Welfare Act was implemented on May 30, 2017, and the modified Emergency Medical Services Act was implemented on December 3, 2017

* *p* -values from the Kruskal–Wallis(K-W); ^+^*p*-values from Dunn’s multiple comparisons test

**Table 3:** Cox proportional hazards regression analysis

(A) Psychiatry department		
Parameter	Age- and sex- adjusted	
	HR (95% CI)	*P* ^*^
Period 1	1	-
Period 2	1.03 (0.91–1.17)	0.60
Period 3	0.91 (0.83–0.98)	< 0.05

(B) Other departments		
Parameter	Age- and sex- adjusted	
	HR (95% CI)	*P* ^*^
Period 1	1	-
Period 2	1.05 (1.01–1.08)	< 0.05
Period 3	1.07 (1.05–1.09)	< 0.05

ED: emergency department; HR: hazard ratio; CI: confidence interval

Period 1 was ED visits before the implementation of the Mental Health Welfare Act. Period 2 was ED visits after the implementation of the Mental Health Welfare Act. Period 3 was ED visits after the implementation of the modified Emergency Medical Services Act

The Mental Health Welfare Act was implemented on May 30, 2017, and the modified Emergency Medical Services Act was implemented on December 3, 2017

* p-values from Cox proportional hazards regression analysis

### Differences in ED LOS for admitted patients before and after the implementation of the mental health welfare act and emergency medical services act

We investigated the impact of the implementation of the MHWA on ED LOS in patients admitted to psychiatry departments. After the implementation of the MHWA, the median ED LOS was 7.4 h in Period 1 and 7.8 h in Period 2, with no statistical difference (*P* = 0.79). However, after the EMSA amendment was implemented, the median ED LOS increased to 9.15 h (Period 1 vs. Period 3, *P* < 0.05) (Table 2A). For patients admitted to non-psychiatric departments, the median ED LOS showed no significant change after the MHWA (Period 1 vs. Period 2: 3.5 vs. 3.4 h, *P* = 0.13). The overall comparison between Period 1 and Period 3 was significant (3.5 vs. 3.4 h, *P* < 0.05), while the median remained unchanged between Period 2 and Period 3 (Table 2B).

**Fig. 2:**
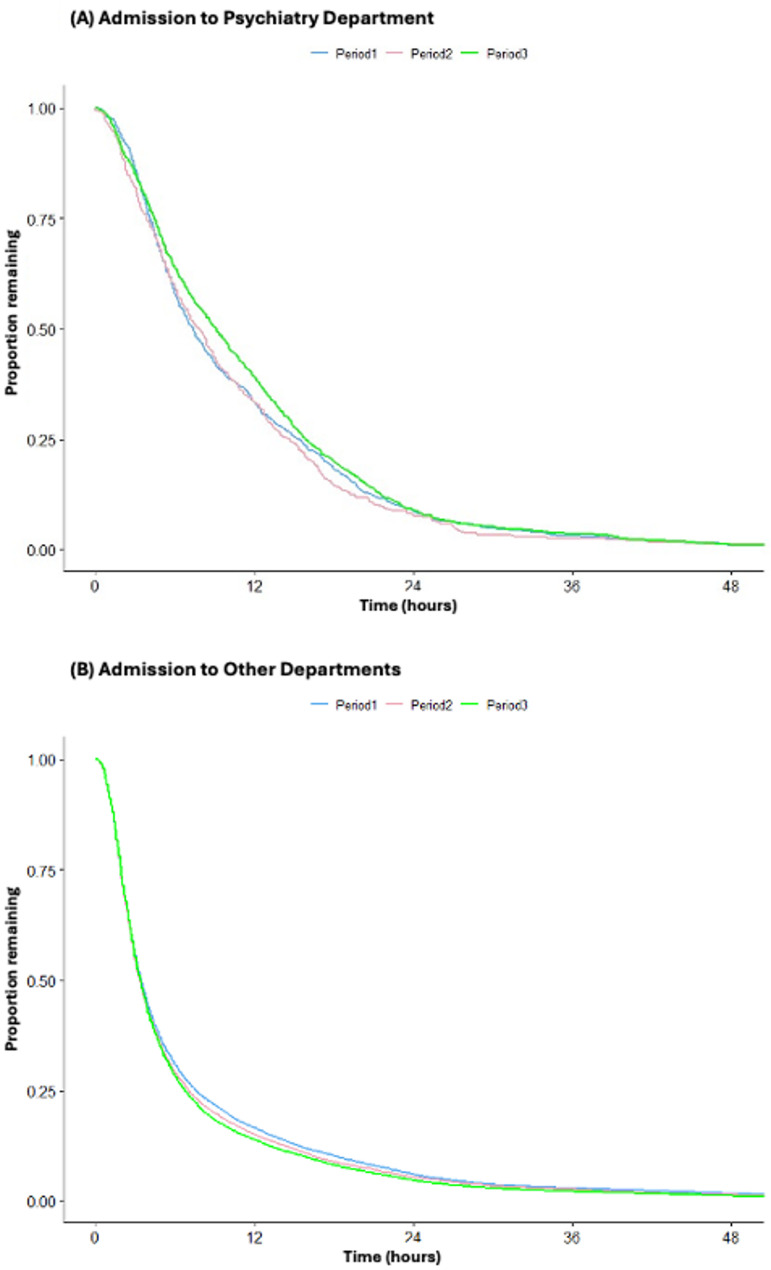
Survival analysis of differences in ED LOS (hours) before and after implementation of the law. (**A**) Admission to psychiatry department, (**B**) Admission to other departments. Period 1 was ED visits before the implementation of the Mental Health Welfare Act. Period 2 was the ED visit after the implementation of the Mental Health Welfare Act. Period 3 was ED visits after the implementation of the modified Emergency Medical Services Act

In Cox regression analysis, adjusted for age and sex, the ED LOS did not change following the implementation of the MHWA (*P* = 0.6). However, it became longer after the implementation of the EMSA (hazard ratio (HR) = 0.91, 95% confidence interval (CI): 0.83 to 0.98, *P* < 0.05) (Fig. 2A, Table 3A). In contrast, for other departments, Cox regression analysis adjusted for age and sex showed shorter ED LOS after implementation of the MHWA (HR = 1.05, 95% CI: 1.01 to 1.08, *P* < 0.05) and the EMSA (HR = 1.07, 95% CI: 1.05 to 1.09, *P* < 0.05) (Fig. 2B, Table 3B).

## Discussion

We investigated the association of the MHWA with ED LOS for suicide attempters admitted to psychiatric departments in Korea. The MHWA alone did not significantly change ED LOS (Period 1 vs. Period 2; Table 3A). A statistically significant increase in ED LOS was observed in Period 3, which followed implementation of both the MHWA and the EMSA amendment (Table 3A). Because these legislative changes occurred within a short time interval, their independent contributions to the observed change in ED LOS cannot be determined.

The MHWA’s tightened compulsory admission criteria may have affected the admission process for suicide attempters. Under the revised law, involuntary admissions extending beyond two weeks require a secondary diagnosis by an external psychiatrist, which involves coordinating visits from public hospital psychiatrists or transporting patients to designated institutions [15, 16]. These procedural requirements may contribute to delays in finalizing admission decisions, potentially prolonging ED stays.

These findings should be interpreted within the broader context of mental health policy reform in Korea. The MHWA was implemented in response to concerns regarding the protection of patient rights, and it strengthened procedural requirements for compulsory admission [14]. Although such regulatory changes may have influenced aspects of clinical decision-making and admission processes in some settings, our results do not provide direct evidence that the MHWA independently prolonged ED LOS.

Patients visiting EDs with mental health concerns have consistently been reported to experience longer ED LOS compared with other emergency patients, reflecting the complexity of care processes, including the need for specialized psychiatric assessment, consultation, and disposition planning [22, 23]. Given that ED LOS is an operational indicator related to throughput and resource allocation, differences in LOS across patient groups may have implications for overall ED performance. Prolonged ED stays have therefore been examined in relation to system efficiency and ED crowding [24]. Policies aimed at limiting ED LOS have been implemented in several countries and were associated with improvements in ED throughput and timeliness of care [25, 26]. In South Korea, the EMSA amendment was implemented on December 3, 2017, in response to concerns about ED crowding, with a policy target of limiting ED LOS to 24 h [21]. 

The care of suicide attempters in the ED often involves collaboration between emergency physicians and mental health professionals [27]. Many studies reported that early intervention by mental health professionals in assessing and treating suicide attempters in the ED played a crucial role in suicide prevention [28]. However, the present study did not evaluate clinical outcomes such as repeat suicide attempts, mortality, or long-term prognosis. Therefore, our findings should not be interpreted as evidence that changes in ED LOS directly influence suicide risk or other patient-centered outcomes.

Broader debates regarding compulsory admission standards, psychiatric bed availability, and suicide prevention strategies have been described in prior studies [29–31]. These issues reflect complex interactions between legal frameworks, healthcare resources, and access to care, which vary across countries. For example, approaches to involuntary admission and emergency psychiatric capacity differ in the United States and Europe [32]. In South Korea, appropriate post-management is provided to medical institutions participating in projects like ED-based management for suicide attempters. However, these efforts have been hampered due to low participation [33], and the lack of standardized guidelines leads to different evaluation and post-management measures among hospitals for suicide attempters. Evaluation of how these broader structural factors influence suicide outcomes requires dedicated investigation beyond the scope of the current study.

There were several limitations to this study. First, since the data were anonymized, each ED visit was treated as an independent case without patient identifiers. This precluded tracking individual patients across visits, making it impossible to analyze repeat ED visits, readmission rates, or subsequent suicide outcomes. Second, as a retrospective observational study, it was not possible to adjust for various factors that may influence ED LOS. Other concurrent changes following the MHWA implementation should also be considered. Lastly, the data transferred to the NEDIS vary depending on the ED level, so only patients visiting centers at or above the center level were included in the study. Furthermore, we could not examine hospital-level or regional variations in ED LOS due to the lack of granular data such as specific hospital identifiers or local psychiatric resources in the national database. Future studies should explore these variations using more detailed clinical registries. Nevertheless, the study data are representative as it was based on the census data of patients visiting national emergency medical centers, and hospitals that include psychiatry as a specialty are large-scale hospitals.

Overall, this study showed that implementation of the MHWA was not associated with a statistically significant immediate change in ED LOS for suicide attempters. Although ED LOS increased during the later study period, the independent contribution of the MHWA cannot be determined due to concurrent policy changes and broader system-level factors. These findings highlight the complexity of evaluating legislative reforms within dynamic emergency care systems. Therefore, legal implementation of mental health welfare measures must consider balancing accessibility to the medical system with individual rights.

## Supplementary Information

Below is the link to the electronic supplementary material.

Supplementary Material 1

## Data Availability

The data that support the findings of this study are available from Korean National Emergency Department Information System, but restrictions apply to the availability of these data, which were used under license for the current study, and so are not publicly available.

## Abbreviations

ED: Emergency Department
LOS: Length of Stay
EMC: Emergency Medical Centers
NEDIS: the National Emergency Department Information System
ICD-10: International Classification of Diseases, Tenth Edition
KTAS: Korean Triage and Acuity System
MHWA: Mental Health Welfare Act
EMSA: Emergency Medical Services Act
