# Supplementary material for: The effect of tightened compulsory admission laws on length of stay in emergency department for suicide attempters
Source: BMC Health Serv Res. 2026 Apr 10;26:719. doi: 10.1186/s12913-026-14474-6 (PMC13192080; doi:10.1186/s12913-026-14474-6)
Supplement: Supplementary file 1 — Supplementary Material 1 [file 12913_2026_14474_MOESM1_ESM.docx]

**
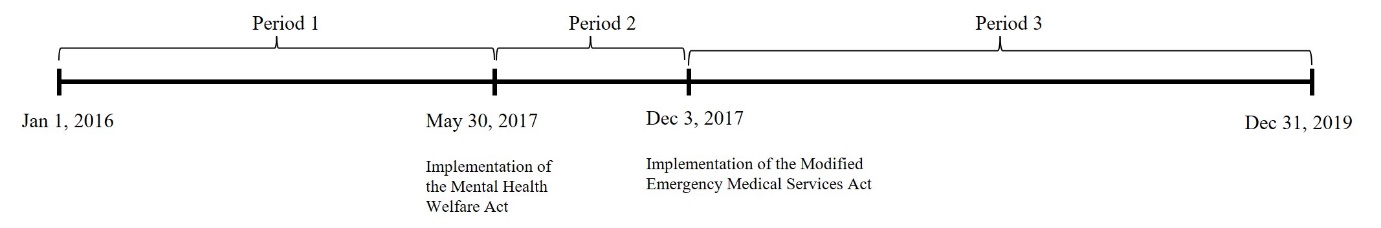
**

**Supplementary Figure 1. Study Groups.** The patients were divided into three groups according to the visit period
